# Development of data for the identification and characterization of proteins found in *Rhodnius prolixus*, *Triatoma lecticularia* and *Panstrongylus herreri*

**DOI:** 10.1016/j.dib.2016.03.053

**Published:** 2016-03-19

**Authors:** Carlos Emmanuel Montandon, Edvaldo Barros, Pedro Marcus Vidigal, Maria Tays Mendes, Ana Carolina Borella Marfil Anhê, Humberto Josué de Oliveira Ramos, Carlo José Freire de Oliveira, Cláudio Lísias Mafra de Siqueira

**Affiliations:** a Department of Biochemistry and Molecular Biology, Federal University of Vicosa, Vicosa, Minas Gerais State, Brazil; b Biomolecules Analysis Center, Federal University of Vicosa, Vicosa, Minas Gerais State, Brazil; c Department of Microbiology, Immunology and Parasitology, Federal University of Triangulo Mineiro, Uberaba, Minas Gerais State, Brazil

**Keywords:** Triatomines, Sialoproteomics, *Rhodnius prolixus*, *Triatoma lecticularia*, *Panstrongylus herreri*

## Abstract

The data presented here were obtained from the saliva of three triatominae, *Rhodnius prolixus*, *Triatoma lecticularia* and *Panstrongylus herreri* from Montandon et al. study, doi:10.1016/j.ibmb.2016.02.009 [3]. These data were obtained from spectra generated by the mass spectrometry of proteins observed through the analysis of 2-D electrophoretic profiles. The data were analyzed according to the UniProt code, protein name, protein group, isoelectric point and molecular weight, electrophoretic profile, molecular mass referring to UniProt, volume percentage referring to the spot of the electrophoretic profile, number of peptides and percent coverage found by mass spectrometry related to the particular proteins. In addition, there characterizations made the most significant protein per spot, and also characterizations made for biological processes and molecular functions for all identified proteins.

**Specifications Table**

**Table:** 

Subject area	Biology
More specific subject area	Biochemistry of macromolecules and entomology
Type of data	Table
How data was acquired	2-D Electrophoresis and Mass spectrometry
Data format	Analyzed
Experimental factors	Purified proteins of triatomine bugs saliva
Experimental features	Acquisition of aspects relating to the spots of the 2-D electrophoretic profiles by mass spectrometry, followed by bioinformatics analysis
Data source location	N/A
Data accessibility	The data are available with this article

**Value of the data**

• The data reveals a set of proteins in the saliva of three species of triatomine (*R. prolixus*, *T. lecticularia* and *P. herreri*), which were characterized by 2-D Electrophoresis, Mass Spectrometry and Treatment of Bioinformatics.
• The data is valuable to the study of correlation with other species of triatomine, or even with other bloodsucking arthropods.
• The data may help to elucidate mechanisms and pathways by which the blood-sucking arthropods perform blood feeding.
• The set of proteins were found provide support in the selection of biomolecules with potential biotechnological use, for example, in the manufacture of drugs.

## 1 Data

The data in Tables 1–3 were acquired by analyzing the spectra related to the spots highlighted in the 2-D protein profiles of the saliva of the triatominae *Rhodnius prolixus*, *Triatoma lecticularia* and *Panstrongylus herreri*.

Table 1 refers to all proteins found in each spot for the three species. It shows the following characteristics for each protein: species, spot related to the electrophoretic profile, UniProt code, protein name, protein group, isoelectric point (pI) and molecular weight (MW) for each electrophoretic profile, molecular mass referring to UniProt, volume percentage referring to the spot of the electrophoretic profile (vol%), number of peptides (#Pep) and percent coverage found by mass spectrometry related to that particular protein.

Table 2 refers to the most significant protein per spot (MSP) for the three species under study. It shows the same characteristics shown in Table 1 for each protein.

Table 3 refers to the proteins found in the studied species according to biological process and molecular function.

## 2 Experimental design, materials and methods

### 2.1 2-D electrophoresis and gel analysis

#### 2.1.1 2-D electrophoresis and gel analysis

The procedure for carrying out the first dimension was performed according to Görg et al. [1] with modifications. We used 7 cm strips of polyacrylamide gel and the isoelectric focusing was maintained for approximately 23 h (300 V for 15 h; 300 Vh in a gradient up to 1000 V; 4000 Vh in a gradient up to 5000 V; 20,000 Vh in a gradient up to 5000 V; 200 V for 10 h).

The second dimension was performed as described by Laemmli [2]. After processing with Coomassie Blue G-250 to highlight the proteins, the gels were digitally photographed using an Image Scanner III device (GE Healthcare, Sweden). The images were calibrated with the Labscan software (GE Healthcare, Sweden). The protein profile images were analyzed with ImageMaster 2D Platinum, version 7.5 (GE Healthcare, Sweden) in order to determine the abundance of protein spots according to the following parameters: smoothness greater than 2, saliency greater than 50, and area greater than 50.

### 2.2 Mass spectrometry

The protein spots with higher vol% were extracted, focusing on greater functional relevance and higher probability of successful identification through the MALDI-TOF/TOF approach.

The proteins contained in the gel fractions were then subjected to the gel trypsinolysis process according to the protocol described by Shevchenko et al. [4]. An aliquot of 1 µL of the solution with the sample (resuspension in 10 µL of acetonitrile 50%/trifluoroacetic acid 0.1%) was applied to a polished steel dish at a ratio of 1:1 in relation to the α-cyano-4-hydroxycinnamic acid matrix. The selected spots were subjected to mass spectrometry in a MALDI-TOF/TOF Ultraflex III device (Bruker Daltonics, Germany), which was managed by version 3.3 of the Flexcontrol software (Bruker Daltonics, Germany). The MS1 data were acquired through the reflective method in the positive mode, with a detection range of 500 to 5000 Da. The MS2 data were acquired through the LIFT method, also in the positive mode. The ions with higher intensity in relation to mass/charge (m/z) were chosen to obtain the MS2 spectra. The resulting spectra were processed using version 3.3 of the FlexAnalysis software (Bruker Daltonics, Germany).

The samples that did not generate results through the MALDI-TOF/TOF analysis, were concentrated again in a speedvac lyophilization device, and then re-suspended in 60 µL of aqueous formic acid 0.1% solution. 10 µL of the solution with the samples was analyzed in the LC-MS Amazon Ion trap mass spectrometer (Bruker Daltonics, Germany) coupled to the UPLC nanoACQUITY system (WATERS, USA), with one trap column and one capillary C18 BEH130 column, and with 100 μm×100 mm −1.7 μm of resolution. The spectrometer operated on the auto-MSn mode and the data for MS1 and MS2 were acquired through the positive mode in a detection range of 300–1500 m/z, and the data for MS2, also in positive mode, were acquired with a detection range of 70–3000 m/z for a standard mass limit of 27%. The acquired data were converted to the mzXML format and processed with version 7 of the Peaks software (Bioinformatics Solutions, Canada) to generate the lists of masses for MS2.

### 2.3 Spectrum analysis and protein characterization

The lists of masses for the spectra of peptides obtained with the MALDI-TOF/TOF and Ion trap LC-MS methods, were analyzed with the MS/MS IonSearch algorithm of version 2.2.07 of the Mascot Daemon software (Matrix Science, UK), and by the Peaks DB, Peaks PTM (post-translational modifications), Spider (mutations), and De novo (De novo sequencing) algorithms of the Peaks software.

Version 3.6.4 of the Scaffold software (Proteome Software, USA) was used to validate the proteins identified by Mascot Daemon, with the identification of both proteins and peptides equal to or above 90%. The Peaks software also validated the proteins identified by the Ion trap LC-MS, using the statistical approach of a False Discovery Rate of less than 1%.

The specific triatomine database built on 12/02/2015 with data of 31,319 proteins of the Triatominae subfamily (Taxonomy ID 70,999), available at the UniProt (UniProt Consortium), was executed on local servers. This database includes the proteins annotated to the genome of R. prolixus and the proteins of the sialotranscritpome of *T. infestans*. The spectrum study used mass variation tolerance values of 0.15 Da and 0.2 Da for the MALDI-TOF/TOF and Ion trap LC-MS data. For the searches and validation, the carbamidomethylation of cysteine residues (alkylation with iodoacetamide) was also used as fixed modification, with the oxidation of methionine being used as the variable modification. For validation, the criterion was used of at least one peptide with a score above 95% and a 95% probability per protein found.

For the characterization of the identified protein spots, we used the following resources: (1) Vol% given by ImageMaster for each spot (which is the percentage of a spot in relation to the total volume of spots) to determine the amount of proteins in the gels and the abundance of each spot; (2) score given by the Scaffold (identity) or Peaks software, number of peptides, coverage and specifications as criteria for the selection of the MSP; and (3) the Gene Ontology functional classification (Gene Ontology Consortium).
